# Effectiveness of the Volunteer Family Connect Program in Reducing Isolation of Vulnerable Families and Supporting Their Parenting: Randomized Controlled Trial With Intention-To-Treat Analysis of Primary Outcome Variables

**DOI:** 10.2196/13023

**Published:** 2019-11-21

**Authors:** Rebekah Grace, Kelly Baird, Emma Elcombe, Vana Webster, Jacqueline Barnes, Lynn Kemp

**Affiliations:** 1 Translational Research and Social Innovation Western Sydney University Liverpool Australia; 2 Institute for the Study of Children, Families and Social Issues Birkbeck University of London London United Kingdom

**Keywords:** volunteer home visiting, randomized controlled trial, families, support services, social relationships, community, Volunteer Family Connect

## Abstract

**Background:**

Volunteer home visiting is a widely adopted community-based approach to support families by linking isolated or vulnerable families with community volunteers who visit their homes weekly over approximately 12 months. This study seeks to robustly evaluate the effectiveness of this model of support for families with young children.

**Objective:**

This paper reports the intention-to-treat analysis of primary and secondary outcomes for a pragmatic randomized controlled trial (RCT) of the Volunteer Family Connect intervention, a volunteer home-visiting program designed to support families with young children who experience social isolation or a lack of parenting confidence and skills.

**Methods:**

The RCT was conducted across seven sites in Australia. Overall, 341 families were recruited: 169 intervention (services as usual+volunteer home visits) and 172 control (services as usual) families. Intervention families received the program for 3-12 months. Participants were invited to complete six data collection points over a 15-month period. Primary outcomes were community connectedness and parenting competence. Secondary outcomes included parent physical and mental health, general parent wellbeing, parent empowerment, the sustainability of family routines, and the parent-child relationship. According to the protocol, the program would be judged to be effective if at least one of the primary outcomes was significantly positive and the other was neutral (ie, intervention families did not demonstrate positive or negative outcomes compared to the control group).

**Results:**

The intervention group demonstrated significant improvement in the primary outcome variable parenting sense of competence as compared to the control group. Overall, there was no significant difference between the intervention and control groups with regard to the primary outcome variable community connectedness, other than on the “Guidance” subscale of the Social Provisions Scale. Because there were statistically significant findings for the total score of one primary outcome variable “parenting sense of competence” and largely neutral findings for the primary outcome variable “community connectedness,” the program met the previously defined criteria for program effectiveness. In relation to secondary outcomes, intervention families reported significantly higher wellbeing and were significantly more likely to feel that life was improving.

**Conclusions:**

The Volunteer Family Connect intervention was considered an effective intervention, with a role to play on the landscape of services available to support vulnerable families with young children.

**Trial Registration:**

Australian New Zealand Clinical Trial Registry ACTRN12616000396426; https://www.anzctr.org.au/Trial/Registration/TrialReview.aspx?id=370304

## Introduction

### Background

Volunteer home visiting is a widely used strategy to support those who are isolated within their communities and require additional support to engage with health and other community services that are available to them and with other local families. Typically, a community volunteer is assigned by a coordinating organization to someone who has been identified as needing social support. The volunteer will visit them on a regular basis, provide general support, and facilitate their engagement with formal services until the individual feels more connected to the community and better able to utilize services independently. Previous research has supported the importance of this less formal, relationship-based approach as complementary to other, more formal services on the service landscape. It is likely to be instrumental in breaking down barriers to service engagement, including language and cultural barriers [1]. Research supports the potential value of volunteer home visits in the distribution of health information [2], support of improved social networks to those who are isolated [3], promotion of emotional wellbeing and parenting competence [4], promotion of positive health outcomes [5], and support of those with chronic illnesses [6].

In the Australian context, volunteer home visiting programs for families with young children have come under threat in recent years, with services reporting the withdrawal of government funding because of the lack of methodologically rigorous evidence demonstrating their effectiveness. The failure to evaluate the effectiveness of this model of support utilizing a robust, gold-standard research design in an Australian context has been mistaken for a lack of program effectiveness. Rigorous trials are required to determine the effectiveness of volunteer home visiting as a form of structured social relationships to support those who are isolated.

### Structured Social Relationships as Intervention

The research evidence demonstrating the importance of social relationships as protective for health and wellbeing is strong. Much of the existing research has emphasized on the role of social networks in the prevention and treatment of mental health disorders such as depression [7,8]. Holt-Lunstad and colleagues [9] refocused attention on biomedical health outcomes, looking specifically at social connection as a risk factor for mortality. They conducted a meta-analytic review and found a 50% increase in the likelihood of survival for participants with strong social relationships. Social isolation was found to place participants at a higher risk of mortality than well-known risk factors, including smoking, excessive drinking, and obesity. Although the prevention of smoking and obesity attracts considerable attention and investment across the world, social relationships are still largely conceptualized as existing within the private realm beyond the scope of service intervention and public health campaigns. However, in recent years, at the level of policy, there has been a growing interest in the importance of social connection. For example, in January 2018, the Prime Minister of the United Kingdom announced the establishment of the Commission for Loneliness [10].

The need to address social connections in the design of service solutions is further supported by research demonstrating rising levels of perceived social isolation and disconnection in the industrialized world. This is largely credited to the increased rates of divorce, separation and single parenthood, geographic mobility, and the decline in extended families living together [11]. In Australia, for example, conservative estimates indicate that 7%-9% of Australians report feeling socially isolated or very isolated, with younger adults being at the highest risk of perceived isolation [12,13].

Addressing social connection through service intervention is not straightforward, because appropriately paid service professionals must maintain professional boundaries and are perceived to provide a service by clients rather than being part of their social network [9]. Nonetheless, increased understanding of the importance of social connection is having a direct impact on health service practice, with a growing emphasis on a relationship-based approach to nursing and allied health care [14]. Establishing support programs run by community volunteers represents an approach to supporting those who are isolated to build social connection within the local community and facilitate engagement with local services [15].

Byrne and colleagues [1] proposed that a holistic approach to family health and wellbeing requires four interconnected arms of support: universal services (eg, primary health care), targeted services (eg, specialist medical services and child protection services), informal networks (eg, friends and families), and structured social support (eg, volunteer “befriending” programs). Organized volunteer programs are clearly not as organic as natural friendships or family ties, but they potentially provide an alternative for people who do not have an informal support network within their local community and may indeed be preferable if there are family tensions. Social networks, structured or organic, play a crucial role in breaking down the barriers to engagement with professional services and in fostering a sense of personal wellbeing [2].

### Structured Social Relationships to Support Parents of Young Children

Parents, especially mothers, are at a high risk of social isolation, particularly in the early years of transition to parenthood when feelings of exhaustion or unpreparedness can be overwhelming [16,17]. In research involving parents with additional challenges, such as having a child with a disability [18], newly arriving in a country [19], or experiencing cognitive or mental health difficulties [20], social isolation is a common theme. A small body of existing research has examined the role of volunteer home visiting programs in supporting improved outcomes for vulnerable families. Collectively, the literature supports the potential value of a home visiting model in contributing to improved outcomes related to maternal emotional wellbeing and an enhanced sense of maternal parenting competence [4] as well as improved family social connectedness [21]. There is evidence to suggest that volunteer home visiting programs can also support child health outcomes such as improved immunization rates and higher rates of exclusive breastfeeding [22].

### The Volunteer Family Connect Effectiveness Trial

This paper describes a pragmatic randomized controlled trial of volunteer home visiting, which was conducted across four states in the east of Australia. The project provides an exemplary model of service collaboration, bringing together three not-for-profit service organizations usually in competition with each other and all independently running volunteer home visiting programs for families with young children (Karitane, The Benevolent Society, and Save the Children Australia). The collaboration, including university research partners and a corporate partner, combined the best elements of the existing programs into one manualized “best practice” program built on research evidence, theoretical underpinning, and practice experience. This program, known as Volunteer Family Connect, was then implemented across all three organizations. Details of program implementation and the research protocol have been published previously [23].

This study addressed two primary outcomes—community connectedness and parenting sense of competence—and compared intervention families (those randomly allocated to receive Volunteer Family Connect in addition to usual care services) with control group families (those randomly allocated to continue to receive usual care services only). In the Australian context, “usual care services” includes free universal health care, government-subsidized early childhood education and care services, and either free or low-cost playgroup or parenting support programs provided by nongovernment organizations varying from one location to another. The control group was therefore still potentially able to access considerable support from within their communities if they sought it out. No restrictions were placed on the intervention group in terms of accessing any additional community support. In fact, this was actively facilitated. Consequently, this study examined the added value of volunteer home visiting within a reasonably comprehensive service context. We hypothesized that intervention families would develop a stronger sense of parenting competence and stronger community support networks than those who continued to receive usual community support services.

## Methods

### Study Design

A pragmatic randomized controlled trial (RCT) was undertaken to rigorously assess the effectiveness of the Volunteer Family Connect intervention in real-world conditions [24]. Supported by the Pragmatic Explanatory Continuum Indicator Summary (PRECIS) tool [24] and in keeping with the “real-world” conditions for a pragmatic randomized trial, this study (1) recruited the full range of families referred (through usual referral pathways) to the Volunteer Family Connect intervention program delivered across the service organizations with no changes to the inclusion/exclusion criteria; (2) compared the volunteer home visiting program with other usual care support services; and (3) tested real-world implementation of the volunteer home visiting program by the service organizations with their current volunteer providers by using guidelines to support quality service provision, but acknowledging that there are variations in practice while rigorously assessing outcomes using standardized measurement tools. The use of the PRECIS tool in supporting the design of this RCT has been reported elsewhere [23].

### Primary Research Question

Is Volunteer Family Connect, a volunteer home visiting intervention, effective in improving the parenting competence and community connectedness of vulnerable families with young children compared with families who receive usual community-based support services?

### Hypothesis

Families receiving a volunteer home visitor will have significantly better family outcomes at program exit (ie, higher sense of parenting competence and stronger community connectedness) than those allocated to continue to receive usual care in the community.

### Secondary Research Question

For the purposes of this paper, results are presented for the secondary research question: Do differences exist in the patterns of parent health, wellbeing, empowerment, parent-child relationship, and family routines over time between those who receive the Volunteer Family Connect program and those in the services as usual control group?

### Participants

#### Eligibility Criteria

Families were assessed against the following eligibility criteria: (1) having one or more children aged 0-5 years, (2) being at-risk of geographic or social isolation, (3) seeking to develop confidence and increase parenting knowledge and skills, (4) residing in the specified service area, and (5) being unable to access resources or other support services. Research participation was supported by the use of interpreters for families with a first language other than English.

In line with usual program practice, families were unable to participate in the study if any of the following conditions applied: (1) active abuse or domestic violence within the family, (2) unmanaged mental illness within the family, (3) substance abuse issues in the family, (4) living in an environment that was unsafe for a volunteer to visit, or (5) under child protection orders or unsettled parenting arrangements. Families who experienced these challenges were referred to more specialized services.

#### Recruitment

**Figure 1:**
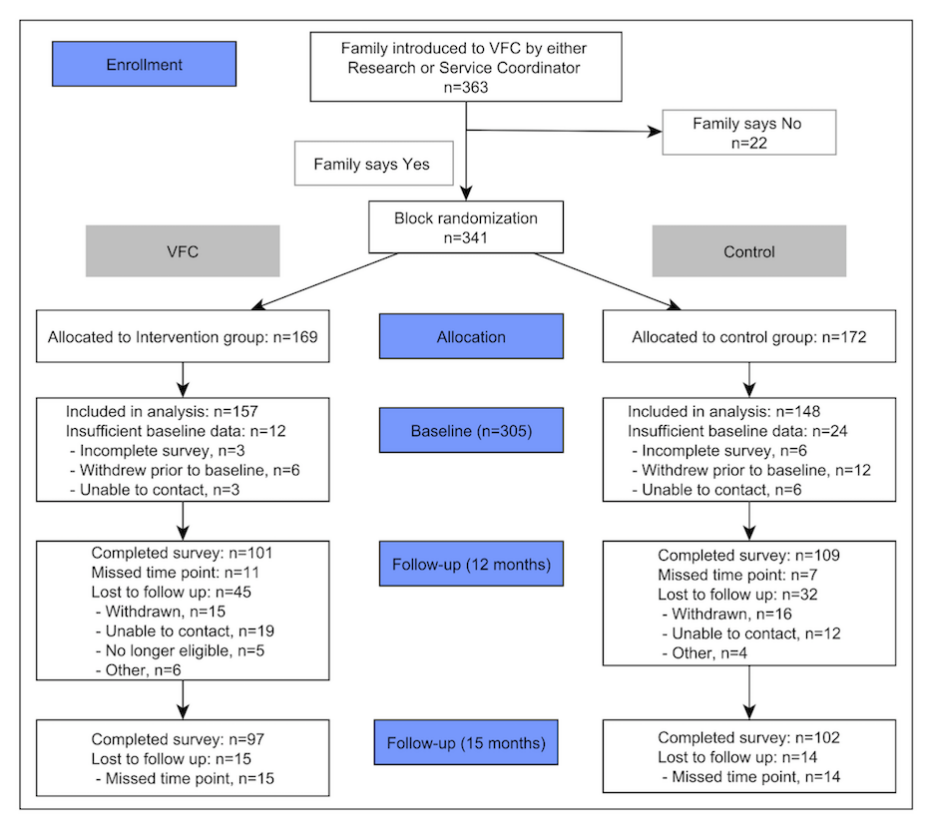
Consolidated Standards of Reporting Trials diagram of participant retention for eligible participants. VFC: Volunteer Family Connect.

Families were recruited to the study either through the usual service referral networks for the Volunteer Family Connect program (eg, child and family health nurses, general practitioners, or family support/social workers) or through self-referral to the program. Families who were eligible to receive the program were invited to speak with a researcher, and if interested, informed consent was secured. Using computer-generated random numbers, the research manager allocated families to the intervention group (services as usual+Volunteer Family Connect) or the control group (services as usual only). The procedure used to recruit and allocate families was described in the study protocol paper [23]. Participant attrition information is provided in Figure 1. At baseline, 341 families were recruited to the study: 169 intervention families (services as usual+Volunteer Family Connect) and 172 control families (services as usual only).

### Intervention

During the trial, the Volunteer Family Connect program was implemented in seven sites across the east of Australia in New South Wales, Queensland, Victoria, and Tasmania. The sites represented city communities (n=2), suburban communities (n=3), and rural communities (n=2).

Families in the intervention group received the Volunteer Family Connect program delivered by one of the three service organizations. Program coordinators matched families with trained community volunteers who visited the family for approximately 2 hours once per week. Volunteers were community members with some experience in working with children, either because of personal parenting experiences or their employment experiences. Volunteers participated in at least 30 hours of training before being matched with a family and underwent police checks to ensure that they could work with children and families. Depending on the needs of the family, volunteers supported families to connect with other services/facilities in their local community as well as modelled positive interactions with children and encouraged parents to identify and meet their personal and family goals.

### Outcome Measures

Table 1 presents the family outcome measures and the associated standardized instrument or, if unavailable, the tool specifically designed for use in this trial. Measurement tools that are widely used within the parenting research literature were selected wherever possible. Measures were presented to families in a single survey instrument, so that all information is parent self-report data. This survey instrument was tested in a feasibility/pilot study in advance of the trial and found to be acceptable to families [25].

**Table 1:** Family and parent outcome measures.

Outcomes measured		Instruments
**Primary outcomes**		
	Parenting competence	Parenting Sense of Competence Scale [26]. All three subscales were collected, including Parenting Satisfaction, Parenting Interest, and Parenting Efficacy. The total score was also calculated.
	Community connectedness	Four Community Connectedness questions were taken from the survey that is used in the Longitudinal Study of Australian Children [27]. Participants were asked to rate, on a 4-point scale, to what extent the following statements were true for them: “If you need information about local services, you know where to find that information.” “You feel a strong sense of identity with your neighbourhood.” “Most people in your neighbourhood can be trusted; you are well informed about local affairs.” Social Provisions Scale [28]. All six subscales were used, including Guidance, Attachment, Opportunity for Nurturance, Social Integration, Reassurance of Worth, and Reliable Alliance.
**Secondary outcomes**		
	Parent physical and mental health	Short Form-12 [29]. This is a short-form 12-item measure, which produces a physical health score and a mental health score.
	Parent enablement	Modified Patient Enablement Instrument [30]. The instrument was modified because the original questions were framed within the health context. The wording was changed for the purposes of this study to apply more generically to the service system.
	General parent wellbeing	The Outcome Rating Scale [31]. On this scale, participants are asked to rate how things have been going for them individually, interpersonally, socially, and overall. Two questions were added about whether life has improved over the last 3 months and whether the parent expects that life will continue to improve.
	Sustainability of family routines	Questions developed based on the Ecocultural Family Interview [32]. Participants were asked to rate family functioning on seven questions relating to family routines (eg, bed time routines, mealtime routines, play time routines, and accessing transport).
	Child-parent relationship	Parental questionnaire (questions from the Canadian National Survey of Parents of Young Children) [33]. Nine questions exploring the parent child relationship (ie, positive/warm parent child interactions and angry/punitive parenting) were taken from the Canadian National Survey. Participants were asked to rate on a 5-point Likert scale the extent to which the events described in the questions happen for them (eg, How often do you and your child laugh together?).

### Data Collection

Families completed a survey every 3 months for 15 months, with a total of six data collection points. The first survey was completed when they were recruited to the study (baseline). The 15-month timeframe took into consideration the differing lengths of time that families remained engaged with the Volunteer Family Connect program (ie, 3-12 months) and allowed for at least one data collection point to take place after exiting from the program.

Wherever possible, baseline surveys were completed face-to-face by a research assistant with families in their home. Following this, they had the option to complete the survey with a member of the research team (ie, at the participant’s home or over-the-phone) or self-complete. Surveys were available as a paper version (ie, pen and paper version completed by hand), an electronic version (Microsoft Word document emailed to the participant), or an online version (Web-based version of the survey using Qualtrics software). In addition, iPads (Apple Inc, Cupertino, California) were used for data collection with families who completed surveys face-to-face with a research assistant using the online Qualtrics (Provo, Utah) version of the survey. All other data (ie, collected on a paper version of the survey) were entered into the Qualtrics survey by a data entry officer.

Data were stored on a password-protected Qualtrics database and backed up to a password-protected folder on a server. Only members of the research team had access to the data. Data were deidentified during data entry, with all names replaced by participant numbers. Storage of data was performed in accordance with the requirements of the Australian National Health and Medical Research Council and the Privacy Act 1988.

### Statistical Analysis

Baseline characteristics were described using mean and SD for scale variables and proportions for categorical data. Statistical comparisons of baseline data were completed using Student *t* test, Mann-Whitney *U*, or Chi-squared test, as appropriate. All analyses were completed using SPSS (version 25.0.0.1; IBM Corp, Armonk, New York).

Linear regression analyses were fitted using a two-way piece-wise regression slope (baseline to 12 months and 12 months to 15 months) to accommodate for the expectation of a nonlinear trajectory. The intention-to-treat regression models were adjusted for site (the stratification variable used for randomization). All regression analyses were completed using the mixed procedure, fitted using the restricted maximum likelihood criterion with the autoregressive one covariance matrix applied to repeated statement [34].

Regression results are reported as mean differences where outcomes have been standardized to mean=0 and SD=1, enabling comparison of outcome measures on different scales. Prior to standardization, data normalization was completed for linear outcomes with nonnormal distributions.

Effect sizes (ESs) were calculated for all regression models. Overall, the program was considered to have been effective if at least one of the primary outcomes was significantly positive and the other was neutral. By “neutral,” we mean that intervention families did not demonstrate a positive or negative outcome in relation to the control group.

### Ethics

Ethical approval for the study was granted by the Macquarie University Human Research Ethics Committee (reference number: 5201401144).

## Results

Participants were recruited between May 2015 and April 2017. Of the 410 participants screened for the intervention, 363 were eligible and 341 consented to be randomized. Of these, 305 completed the baseline survey and were enrolled into the trial. At 12 months, 228 (75%) women completed the follow-up survey.

Baseline demographic characteristics are reported by randomization group in Table 2. At baseline, there were no statistical differences between the intervention and control groups in any of these characteristics.

All primary outcome measures showed increasing scores over the duration of the intervention, indicating improvements in parenting sense of competence and community connectedness including social provisions,. Between baseline and 12 months, participants receiving the Volunteer Family Connect program improved significantly more than those in the control group in their parenting sense of competence (F_367.6_=11.2, *P*=.003). In addition, participants receiving the Volunteer Family Connect program had a significantly improved outcome on the Guidance subscale of the Social Provisions Scale (F_1122.6_=4.07, *P*=.04; Table 3). Findings were not significant for the other subscales of the Social Provisions Scale or for the Community Connectedness scale.

**Table 2:** Demographic characteristics of intervention and control families.

Demographic variable		Total (n=305)			Intervention (n=157)			Control (n=148)	
		Retained^a^ (n=228)	Lost (n=77)	Retained (n=112)		Lost (n=45)	Retained (n=116)		Lost (n=32)
**Categorical variable, n (%)**									
	Mother’s education less than year 12	40 (17.5)	11 (14.3)	17 (15.2)		6 (13.3)	23 (19.8)		5 (15.6)
	Culturally and linguistically diverse	50 (21.9)	25 (32.5)	27 (24.1)		14 (31.1)	23 (19.8)		11 (34.4)
	High support needs^b^ for participant	44 (19.3)	12 (15.6)	20 (17.9)		8 (17.8)	24 (20.7)		4 (12.5)
	High support needs for other adult in house	11 (4.8)	2 (2.6)	5 (4.5)		2 (4.4)	6 (5.2)		0 (0.0)
	High support needs for child in house	57 (25.0)	17 (22.1)	24 (21.4)		9 (20.0)	33 (28.4)		8 (25.0)
	High support needs for person in house at baseline	62 (27.2)	18 (23.4)	28 (25.0)		10 (22.2)	34 (29.3)		8 (25.0)
	High support needs for person in house at any stage in program	91 (39.9)	19 (24.7)	44 (39.3)		11 (24.4)	47 (40.5)		8 (25.0)
	Aboriginal or Torres Strait islander	11 (4.8)	4 (5.2)	8 (7.1)		2 (4.4)	3 (2.6)		2 (6.3)
**Scale, mean (SD)**									
	Mother’s age	34.1 (6.8)	32.1 (5.5)	34.3 (6.7)		32.6 (5.8)	33.9 (6.9)		31.5 (5.0)
	Adults living in household	0.9 (0.6)	0.9 (0.6)	0.9 (0.6)		0.8 (0.6)	0.9 (0.7)		0.9 (0.6)
	Children living in household	2.2 (1.2)	2.1 (1.0)	2.0 (1.1)		2.1 (1.1)	2.3 (1.2)		2.1 (0.9)

^a^Retained indicates participation to at least 12 months. Participants were also interviewed at 15 months during the postintervention period.

^b^“High Support Needs” refers to a diagnosed disability, chronic health condition, or mental health condition.

**Table 3:** Results of univariate regression demonstrating change from baseline to 12 months, comparing intervention (Volunteer Family Connect) and control families. Descriptive statistics report the sample size, mean, and SD of each outcome measure at 12 months.

Outcomes			Descriptive statistics				Comparison statistics (baseline to 12 months)						
			Intervention		Control		Statistic^a^	Effect over 3 months^b^		Adjusted 95% CI		*P* value	
			N	Mean (SD)	N	Mean (SD)							
**Primary outcomes**													
	Parenting sense of competence		100	4.10 (0.67)	107	3.99 (0.75)	0.198	0.109		0.04 to 0.18		<.001^c^	
	Community connectedness		101	14.85 (2.69)	109	13.90 (3.14)	–0.007	0.03		–0.04 to 0.10		.41	
	**Social provisions scale**												
		Guidance	101	13.14 (2.14)	109	12.94 (2.17)	0.157	0.076		0.01 to 0.14		.03^c^	
		Reassurance of worth	99	12.01 (1.99)	109	12.18 (2.01)	0.016	0.018		–0.05 to 0.09		.62	
		Social integration	100	12.56 (1.91)	109	12.07 (1.98)	0.148	0.06		–0.01 to 0.13		.08	
		Attachment	101	12.41 (2.38)	109	12.24 (2.27)	0.121	0.06		–0.01 to 0.13		.09	
		Opportunity for nurturance	101	13.61 (2.14)	109	13.92 (1.86)	–0.081	–0.032		–0.10 to 0.04		.38	
		Reliable Alliance	100	13.26 (1.98)	109	12.95 (2.13)	0.023	0.043		–0.02 to 0.11		.20	
**Secondary outcomes**													
	**Short Form-12**												
		Physical	101	47.90 (9.53)	109	47.80 (9.93)	–0.052	–0.016		–0.08 to 0.05		.65	
		Mental	101	44.37 (9.88)	109	45.1 (12.24)	0.123	0.039		–0.03 to 0.11		.27	
	Parent enablement		97	5.27 (3.94)	109	4.50 (3.83)	0.114	0.057		–0.01 to 0.13		.11	
	Life in general		98	0.86 (0.73)	109	0.78 (0.72)	0.104	–0.019		–0.09 to 0.05		.60	
	Outcome rating scale		101	28.81 (7.22)	109	27.1 (7.55)	0.096	0.069		0.00 to 0.14		.04^c^	
	Has life improved in the previous 3 months?		101	7.20 (2.26)	109	6.64 (2.21)	0.197	0.069		0.00 to 0.14		.04^c^	
	Do you think life will improve in the next 3 months?		100	8.05 (1.77)	108	7.64 (1.72)	0.035	0.056		–0.01 to 0.12		.10	
	**Family routines**												
		Getting out of the house	100	4.33 (2.84)	109	4.60 (3.01)	0.061	0.014		–0.05 to 0.08		.68	
		Access to transport	101	2.23 (2.24)	109	2.61 (2.70)	–0.122	–0.037		–0.11 to 0.03		.29	
		Time for tasks	101	5.61 (2.73)	109	5.64 (2.77)	0.062	0.014		–0.05 to 0.08		.68	
		Time with child	100	7.89 (2.33)	109	7.45 (2.42)	0.196	0.064		0.00 to 0.13		.06	
		Meal-time routine	101	7.87 (2.47)	109	7.38 (2.52)	0.175	0.053		–0.01 to 0.12		.12	
		Bed-time routine	101	7.90 (2.32)	109	7.75 (2.35)	0.174	0.049		–0.02 to 0.12		.19	
		Manage day-to-day tasks	101	6.96 (2.15)	108	6.94 (2.45)	0.153	0.053		–0.02 to 0.12		.13	
	**Parent-child relationship**												
		Warmth	101	17.15 (2.27)	109	16.83 (2.18)	0.017	0.013		–0.06 to 0.09		.72	
		Angry	101	11.0 (3.07)	109	10.83 (3.09)	–0.064	–0.017		–0.09 to 0.06		.66	

^a^Comparative statistic is the mean difference (intervention minus control) of the outcome measure after data normalization and standardization.

^b^Effect over 3 months represents change between Volunteer Family Control and control groups in standardized score during each 3-month period (estimated β).

^c^Significant results.

**Figure 2:**
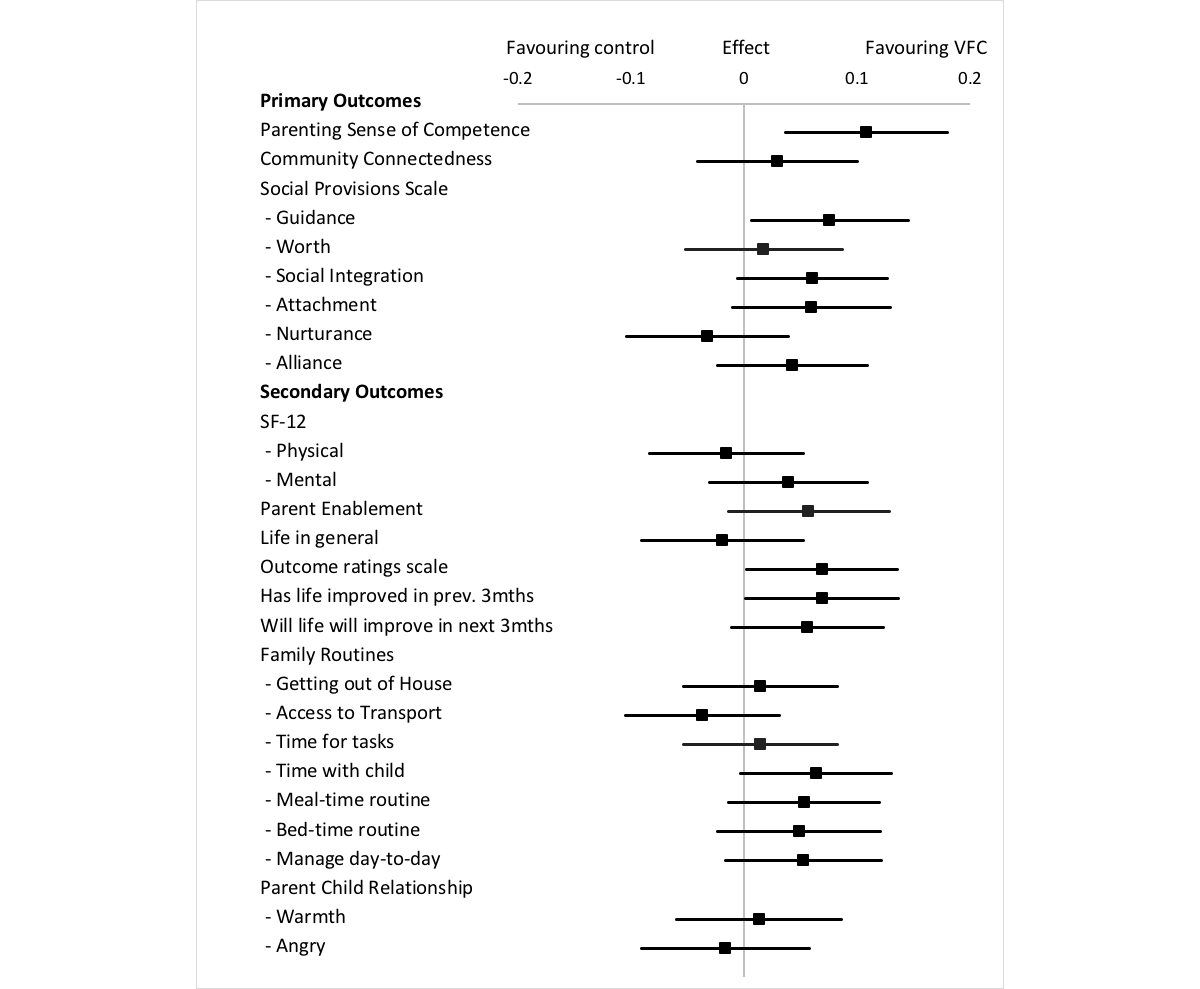
Effect of intervention on outcome measures – baseline to 12 months. Effect represents change between Volunteer Family Connect and control groups in standardized score during each 3-month period, Est β. VFC: Volunteer Family Connect; SF: Short Form.

Regarding the secondary outcomes, participants in the intervention group rated their individual, interpersonal, and social lives as significantly better at 12 months after baseline than control participants (F_446.4_=4.10, *P*=.04; Table 3). There were no statistically significant changes for the intervention group families compared to the control group families in parenting style, parent enablement, physical health, or mental health over the course of the intervention. There was a trend toward improvements (more time: *P*=.06, regular meals: *P*=.12, regular bed times: *P*=.19, life has got better: *P*=.04, and life will continue to get better: *P*=.10) in outcomes of family routines and life, including having more time to spend with their child, having more regular meal times and bed times, and feeling that life was getting better and would continue to get better (Table 3). The forest plot in Figure 2 presents the standardized change score and 95% CI for each variable. Multimedia Appendix 1 reports the full univariate outcome models.

## Discussion

### Principal Findings

The findings from this study met the criteria for program effectiveness defined in our previous peer reviewed protocol paper [23]. To be considered effective, the program needed to achieve positive results on at least one of the primary outcomes, with the other primary outcome achieving at least neutral results. A strong statistically significant finding was improved outcomes for the intervention group on the Parenting Sense of Competence Scale. However, the results for the Social Provisions Scale (SPS) were mixed. The SPS was a key measure of community connectedness, and only one subscale (Guidance) on this measure demonstrated a significant finding, indicating that families in the intervention group were more likely to report that there was someone in their life they could go to for advice and information. Nevertheless, the forest plot presenting effect sizes (Figure 2) shows positive trends for the intervention group on three additional SPS subscales, including Social Integration (sense of belonging to a group), Attachment (emotional closeness with another person), and Reliable Alliance (having someone who could be counted on in times of stress).

There were some significant findings among the secondary outcomes measured. General parent wellbeing, as measured by the Outcome Rating Scale, was significantly higher for the parents who received the Volunteer Family Connect program. Volunteer Family Connect program parents were also significantly more likely to report that life had improved in the last 3 months, and there was a trend toward believing that life will continue to improve. Positive trends were present throughout the analysis, including a clear trend of improvement in parent enablement for the intervention families (*P*=.11). Of the seven variables designed to measure the sustainability of family routines, four showed improvement for the intervention families. Those who received the Volunteer Family Connect program were more likely to feel that they were spending more time with their children, had established sustainable meal and bedtime routines, and were managing day-to-day tasks more effectively. Our findings did not demonstrate differences between the intervention and control families on measures of health or parent-child relationship. Importantly, however, there were no significant impacts for the control group over the intervention group.

Addressing the complexity of need that exists within communities in Australia requires a continuum in the range of services available: A rich service landscape that is responsive in the early identification of support needs and meaningful within the local community context [35]. The findings presented in this paper support the effectiveness of structured social relationships, in the form of a volunteer home visiting program, in improving outcomes for isolated or marginalized families with young children. The study contributes to the early intervention and prevention literature, providing evidence for the potential for communities to mobilize as an intervention force in addressing social isolation as a risk factor for vulnerable families [36]. Although the intervention families increased in their connection to community over the course of this research, so did the control group, resulting in nonsignificant findings on our measures of community connectedness for all but the Guidance subscale of the Social Provisions Scale. Our participant groups were predominantly recruited through referral from existing community services, and therefore, these findings may reflect some bias within the sample in that participants had at least some level of connection to community prior to the trial. A review of recruitment strategies to the program may be important to ensure that volunteer support is available to those families who experience significant isolation.

This study was limited by the relatively small participant numbers, and it may be that some of the trends evident within the data would have reached significance with a larger sample size. Another limitation was that it was not possible to mask the group allocation of the participants for the data collection team. Although researchers were blind at the outset, participants disclosed this information in their responses to questions about their experiences with services. This intention-to-treat analysis did not include analyses of benefit of the program for families receiving a longer or shorter duration of intervention, the characteristics of families who may be more or less likely to benefit from this volunteer home visiting intervention, or the relationship between family outcomes and the fidelity of program delivery. These important analyses will be conducted and will be published in subsequent papers, providing an opportunity to explore greater precision in the targeting and provision of the Volunteer Family Connect program.

### Conclusions

The findings from this pragmatic randomized controlled trial examining the effectiveness of the Volunteer Family Connect program demonstrated significant findings in one of the primary outcome, parenting sense of competence, and mixed findings in the other primary outcome community connectedness. The results suggest that high-quality volunteer home visiting programs such as Volunteer Family Connect, with volunteers given training, guidance, and supervision, have a role to play in the landscape of services designed to support families with diverse needs—a role that is complementary to formal service provision and strengthens the parenting confidence, wellbeing, and optimism of vulnerable families.

## Appendix

Multimedia Appendix 1 Tests of fixed effects.

Multimedia Appendix 2 CONSORT 2010 statement.

## Abbreviations

ES: effect size
PRECIS: Pragmatic Explanatory Continuum Indicator Summary
RCT: randomized controlled trial
SPS: Social Provision Scale
