# Supplementary material for: Effectiveness of the Volunteer Family Connect Program in Reducing Isolation of Vulnerable Families and Supporting Their Parenting: Randomized Controlled Trial With Intention-To-Treat Analysis of Primary Outcome Variables
Source: JMIR Pediatr Parent. 2019 Nov 21;2(2):e13023. doi: 10.2196/13023 (PMC6895872; doi:10.2196/13023)
Supplement: Multimedia Appendix 1 [file pediatrics_v2i2e13023_app1.pdf]

Appendix

Table 1: Tests of Fixed effects

		Cohort		Site		Baseline to 12m		12m to 15m		Cohort * Baseline to 12m		Cohort * 12m to 15m	
**Primary Outcome measure**		**F**	**p**	**F**	**p**	**F**	**p**	**F**	**p**	**F**	**p**	**F**	**p**
Parenting sense of Competence		0.07	0.79	1.69	0.12	43.17	0.00	0.75	0.39	8.86	0.00	0.98	0.32
Community Connectedness		0.04	0.85	2.96	0.01	72.12	0.00	0.01	0.91	0.67	0.41	1.69	0.19
Social Provisions scale	Guidance	0.04	0.84	0.79	0.58	31.52	0.00	1.75	0.19	4.58	0.03	4.07	0.04
	Reassurance of Worth	0.00	0.97	0.20	0.98	10.43	0.00	0.49	0.48	0.25	0.62	0.44	0.51
	Social Integration	0.06	0.81	0.93	0.48	26.80	0.00	0.29	0.59	3.17	0.08	0.05	0.83
	Attachment	0.03	0.86	1.24	0.29	14.11	0.00	0.17	0.68	2.82	0.09	1.13	0.29
	Opportunity for Nurturance	0.03	0.87	1.20	0.30	5.65	0.02	1.26	0.26	0.79	0.38	0.98	0.32
	Reliable Alliance	0.13	0.72	1.25	0.28	16.68	0.00	0.59	0.44	1.61	0.20	0.15	0.70
**Secondary outcome measures**													
SF-12	Physical	0.25	0.61	1.09	0.37	7.12	0.01	0.34	0.56	0.21	0.65	0.01	0.94
	Mental	0.19	0.66	1.24	0.28	25.16	0.00	2.30	0.13	1.21	0.27	0.12	0.73
Parent Enablement		0.01	0.92	0.42	0.87	48.34	0.00	0.00	0.97	2.52	0.11	0.11	0.74
Life in general		0.05	0.82	1.10	0.37	22.00	0.00	0.65	0.42	0.27	0.60	0.82	0.37
Outcome rating Scale		0.05	0.82	1.23	0.29	47.22	0.00	0.00	0.96	4.10	0.04	0.01	0.94
Has life improved in the previous 3 months		0.40	0.52	0.88	0.51	25.85	0.00	0.06	0.80	4.05	0.04	1.03	0.31
Do you think life will Improve in the next 3 months		0.35	0.56	0.86	0.52	8.33	0.00	0.05	0.82	2.71	0.10	0.67	0.41
Family Routines	Getting out of house	0.24	0.62	1.97	0.07	39.92	0.00	2.45	0.12	0.17	0.68	0.60	0.44
	Access to transport	0.09	0.76	3.02	0.01	4.70	0.03	3.17	0.08	1.13	0.29	0.04	0.83
	Time for tasks	0.06	0.80	1.53	0.17	12.40	0.00	0.85	0.36	0.17	0.68	0.33	0.57
	Time with child	0.35	0.56	0.61	0.72	0.72	0.40	1.39	0.24	3.50	0.06	1.98	0.16
	Meal-time routine	0.15	0.69	0.82	0.55	3.59	0.06	0.66	0.42	2.45	0.12	0.71	0.40
	Bed-time routine	0.34	0.56	0.76	0.60	0.52	0.47	0.02	0.90	1.75	0.19	0.22	0.64
	Manage day-to-day	0.03	0.86	1.80	0.10	6.36	0.01	0.52	0.47	2.26	0.13	0.44	0.51
Parent Child relation-ship	Warmth	0.01	0.91	1.24	0.29	6.41	0.01	0.05	0.82	0.13	0.72	0.83	0.36
	Angry	0.03	0.87	1.70	0.12	0.08	0.77	4.50	0.03	0.20	0.66	0.04	0.84
